# ToSkORL: Selbst- und Fremdeinschätzung bei der Untersuchung des Kopf-Hals-Bereichs

**DOI:** 10.1007/s00106-021-01097-y

**Published:** 2021-10-07

**Authors:** Kariem Sharaf, Axelle Felicio-Briegel, Magdalena Widmann, Johanna Huber, Tanja Kristina Eggersmann, Ursula Stadlberger, Florian Schrötzlmair, Martin Canis, Axel Lechner

**Affiliations:** 1 Klinik und Poliklinik für Hals‑, Nasen- und Ohrenheilkunde, LMU Klinikum, Marchioninistraße 15, 81377 München, Deutschland; 2 Institut für Didaktik und Ausbildungsforschung in der Medizin, LMU Klinikum, Pettenkoferstraße 8a, 80336 München, Deutschland; 3 Klinik und Poliklinik für Frauenheilkunde und Geburtshilfe, LMU München, Marchioninistraße 15, 81377 München, Deutschland

**Keywords:** Studentische Selbsteinschätzung, Medizinische Ausbildung, HNO-Untersuchung, Medizinstudium, Lehrevaluation, Student self-assessment, Medical education, ENT examination, Medical training, Teaching evaluation

## Abstract

**Hintergrund:**

Ein zentrales Ziel des Medizinstudiums ist der Erwerb theoretischer und praktischer Kompetenzen. Es mangelt jedoch an Evidenz, wie der Erwerb von Kompetenzen in speziellen Untersuchungstechniken gemessen werden kann. ToSkORL (Teaching of Skills in Otorhinolaryngology) ist ein Projekt, das die studentische Selbstwahrnehmung ihrer Kompetenz bei speziellen Untersuchungstechniken der Hals-Nasen-Ohren-Heilkunde und des Kopf-Hals-Bereichs aus didaktisch-wissenschaftlicher Sichtweise beleuchtet.

**Methodik:**

Im Rahmen des Untersuchungskurses erfolgte eine standardisierte mündlich-praktische Prüfung zu neun verschiedenen Untersuchungstechniken. Vor der Prüfung erfolgte eine Evaluation der studentischen Selbsteinschätzung mittels Fragebogen, die Prüfung wurde mittels Checkliste durch die Prüfenden standardisiert geprüft. Selbst- und Fremdeinschätzung nach der Likert-Skala wurden korreliert. Die neun Untersuchungstechniken wurden jeweils 42-mal von insgesamt 91 Studierenden in gegenseitiger Untersuchung durchgeführt.

**Ergebnisse:**

Die Selbsteinschätzung der Kompetenz in den Untersuchungstechniken variiert erheblich, insgesamt schätzten Studierende ihre eigene Untersuchungskompetenz weitgehend unabhängig von Alter und Geschlecht meist realistisch ein. Studierende mit einem hohen Interesse an der Hals-Nasen-Ohren-Heilkunde gaben bessere Selbsteinschätzungen an, neigten jedoch auch eher zur Selbstüberschätzung. Bei Untersuchungen des mittleren Schwierigkeitsniveaus ergab sich die größte Divergenz von Selbst- und Fremdeinschätzung.

**Schlussfolgerung:**

Die studentische Selbsteinschätzung ist ein geeignetes Instrument zur Messung der Untersuchungskompetenz in der Hals-Nasen-Ohren-Heilkunde. Es sollte ein besonderer Fokus auf die Lehre vermeintlich mittelschwerer Untersuchungstechniken gelegt werden, da diese am stärksten über- und unterschätzt werden.

**Zusatzmaterial online:**

Die Online-Version dieses Beitrags (10.1007/s00106-021-01097-y) enthält zwei Mini-Clinical-Exam(CEX)-Evaluationsbögen für Studenten und Untersucher. Beitrag und Zusatzmaterial stehen Ihnen auf www.springermedizin.de zur Verfügung. Bitte scannen Sie den QR-Code, das Zusatzmaterial finden Sie beim Beitrag unter „Ergänzende Inhalte“.

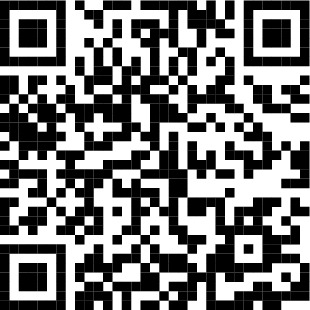

QR-Code scannen & Beitrag online lesen

## Abstract (en)

**Background:**

A central goal of medical school is acquisition of theoretical and practical competences. However, evidence on how capacity acquisition can be measured for special examination techniques is scarce. ToSkORL (Teaching of Skills in Otorhinolaryngology) is a project aimed at scientifically and didactically investigating students’ self-evaluation skills in otorhinolaryngologic and head and neck examination techniques.

**Methods:**

During the examination techniques course, a standardized oral and practical exam for nine different techniques was conducted. Using Likert scales, self-evaluation was based on questionnaires before the clinical skills exam and objective evaluation was performed by the examiners during the examination using a checklist. Self- and objective evaluation were correlated. Nine different examination skills were assessed 42 times each by a total of 91 students.

**Results:**

Self-evaluation of competence in the different examination skills varied widely. Nevertheless, self- and objective evaluation correlated well overall, independent of age and gender. Students highly interested in otorhinolaryngology rated their own skills higher but tended toward overestimation. For examination items with intermediate difficulty, the highest divergences between self- and objective evaluation were found.

**Conclusion:**

Student self-evaluations are an appropriate instrument for measuring competences in otorhinolaryngologic examinations. Instructors should focus on items with allegedly intermediate difficulty, which are most often over- and underestimated.

Der Kompetenzerwerb in praktischen Fertigkeiten ist ein zentrales Ziel des Medizinstudiums und steht auch in der Neufassung des Nationalen Kompetenzbasierten Lernzielkatalogs Medizin (NKLM) besonders im Fokus. Die Vermittlung der speziellen Untersuchungstechniken der Hals-Nasen-Ohren-Heilkunde und die erfolgreiche „Messung“ von Kompetenzerwerb bleiben große Herausforderungen an den medizinischen Fakultäten.

## Hintergrund

Die praxisorientierte Ausbildung und das frühzeitige Erlernen klinisch-praktischer Fähigkeiten zum ärztlichen Kompetenzerwerb haben in den letzten Jahrzehnten einen immer größeren Stellenwert im klinischen Studienabschnitt des Medizinstudiums bekommen [1]. Auch in der Hals-Nasen-Ohren-Heilkunde nimmt der Praxisunterricht einen sehr wichtigen Platz in der studentischen Lehre ein. Dennoch zeigten mehrere Studien unter Medizinern kurz nach Studienabschluss, dass sie sich in wichtigen Bereichen, die den Einsatz praktischer Fertigkeiten verlangen, nicht ausreichend auf den Berufsalltag vorbereitet fühlen [2, 3]. Dies konnte unabhängig vom individuellen theoretischen Wissen festgestellt werden. Das Erlernen praktischer Fähigkeiten sowie das entsprechende Selbstvertrauen, diese einzusetzen, sind jedoch eine wichtige Grundlage für die klinische Tätigkeit. Auf der anderen Seite sollte die Einschätzung der eigenen Fähigkeiten möglichst realitätsnah sein. Ein Unterschätzen könnte ebenso zu einer Fehldiagnose führen wie ein Überschätzen der Kompetenzen patientengefährdend sein könnte. Auf ein gefährliches Überschätzen ohne das Bewusstsein dafür weisen Hodges et al. hin [4]. Eine Metaanalyse zu verschiedenen Aspekten klinisch-praktischer Fertigkeiten zeigte eine in verschiedenen Kompetenzbereichen moderat ausgeprägte Fähigkeit zur Selbsteinschätzung [5].

Als wichtiger Fachdisziplin des Kopf-Hals-Bereichs mit enger Vernetzung zu vielen anderen Fachrichtungen wie zum Beispiel der Neurologie und Pädiatrie dienen Untersuchungstechniken der Hals-Nasen-Ohren-Heilkunde der Diagnostik einer Vielzahl von Symptombildern und Erkrankungen. Während beispielsweise Studien zur Einschätzung operativer Fähigkeiten in der Hals-Nasen-Ohren-ärztlichen Weiterbildung existieren, gibt es nur wenige Studien zur Vermittlung der basalen Untersuchungstechniken des Fachgebiets im Medizinstudium [6–10].

Das Projekt ToSkORL (Teaching of Skills in Otorhinolaryngology) an der LMU München soll zum einen die Selbst- sowie Fremdeinschätzungen zur Kompetenz bei ausgewählten Untersuchungstechniken im Rahmen einer mündlich-praktischen Prüfung in Hals-Nasen-Ohren-Heilkunde während des Medizinstudiums darlegen und zum anderen die Abweichung von Selbst- und Fremdeinschätzung als Maß zur Fähigkeit der realistischen Selbstwahrnehmung beurteilen.

## Methodik

### Lehrkonzept

Die Lehre zu Untersuchungstechniken des Kopf-Hals-Bereichs erfolgt im klinischen Abschnitt des Medizinischen Curriculums München (MeCuM), dem Humanmedizinstudiengang der LMU München, in weit überwiegendem Maß durch die Klinik und Poliklinik für Hals-Nasen-Ohren-Heilkunde. Einzelne Untersuchungstechniken wie beispielsweise die zervikale Lymphknotenuntersuchung werden jedoch bereits im Rahmen eines fächerübergreifenden Longitudinalkurses behandelt. Alle Studierenden belegen obligat im Rahmen des Studiums zwischen dem zweiten und vierten klinischen Semester zuerst einen Propädeutik-Kurs, den sog. Spiegelkurs, dem frühestens im vierten und spätestens im sechsten klinischen Semester Untersuchungskurse auf den Stationen und in der Poliklinik im Rahmen des Bedside-Teachings folgen. Fakultativ ergänzt werden diese Kurse durch ein klinisches Wahlpflichtfach, Kurse in ambulanter Medizin sowie Möglichkeiten zur Famulatur und der Ableistung eines Tertials des praktischen Jahres. Im selben Semester wie die Bedside-Teachings und zur Vermittlung des theoretischen Wissens erfolgen zudem drei Vorlesungen, fünf Seminare und ein Repetitorium sowie zwei „Problem-Based-Learning-Tutorials“.

Die umfassende, zielgerichtete Vermittlung der im Nationalen Kompetenzbasierten Lernzielkatalog Medizin von 2015 (NKLM 2015) vorgesehenen Untersuchungstechniken mit Bezug zur Spiegeluntersuchung im HNO-Bereich erfolgt während des Bedside-Teachings [1]. Am letzten Kurstermin findet eine mündlich-praktische Prüfung statt, deren Bestehen Voraussetzung für den Scheinerwerb ist. Die Liste der zu erlernenden Untersuchungstechniken ist in Tab. 1 (linke Spalte) dargestellt.

**Table 1:** 

	Mini-Clinical-Exam-Modul 4	ToSkORL OSCE
*Ohr und Gleichgewichtsorgan*	Erhebung eines Trommelfellbefunds mit Handotoskop oder Ohrmikroskop	Erhebung eines Trommelfellbefunds mit Handotoskop
	Durchführung der Stimmgabeltests nach Weber und Rinne	Erhebung eines Trommelfellbefunds mit Ohrmikroskop
	Orientierende Gleichgewichtsuntersuchung inkl. Nystagmusprüfung mit Frenzel-Brille und Lagerungsproben	Durchführung der Stimmgabeltests nach Weber und Rinne
*Nase und Nasennebenhöhlen*	Erhebung eines Nasenhaupthöhlenbefunds mittels anteriorer Rhinoskopie	Erhebung eines Nasenhaupthöhlenbefunds mittels anteriorer Rhinoskopie
		Erhebung eines Nasennebenhöhlen- und Nasopharynxbefunds mittels 30°-Optik
*Mundhöhle, Rachen und Kehlkopf*	Erhebung eines Oropharynxbefunds	Erhebung eines Mundhöhlen- und Oropharynxbefunds inklusive Untersuchung der Speicheldrüsen und Hirnnerven IX und XII
	Klinische Untersuchung der großen Kopfspeicheldrüsen und ihrer Ausführungsgänge	Erhebung eines Kehlkopfbefunds mittels 70°- oder 90°-Optik inklusive Untersuchung des Hirnnerven X
	Klinische Untersuchung der Hirnnerven IX und XII	
*Äußerer Kopf-Hals-Bereich*	Erhebung des Halslymphknotenstatus	Erhebung des Halslymphknotenstatus
	Klinische Untersuchung bei Verdacht auf zentrale Mittelgesichtsfraktur	Klinische Untersuchung bei Verdacht auf zentrale Mittelgesichtsfraktur und akute Rhinosinusitis inklusive Untersuchung des Hirnnervs V
	Klinische Untersuchung bei Verdacht auf akute Rhinosinusitis	
	Prüfung und Graduierung der Fazialisfunktion	
	Klinische Untersuchung der Hirnnerven V und XI	

### Prüfungsmodalitäten und Checkliste

Nach Absolvieren der Bedside-Teachings schätzten die Studierenden mittels einer fünfstufigen Likert-Skala („trifft voll zu“ bis „trifft gar nicht zu“) selbst ein, in welchem Ausmaß sie die einzelnen Untersuchungstechniken des Kopf-Hals-Bereichs beherrschten. Zusätzlich wurden Alter, Geschlecht, Semesterzahl und Fragen zum Interesse am Fachgebiet der Hals-Nasen-Ohren-Heilkunde erhoben. Studierende mit weniger als drei absolvierten Bedside-Teachings zum Zeitpunkt der Prüfung wurden ausgeschlossen.

Statt der im Curriculum vorgesehenen mündlich-praktischen Mini-CEX-Prüfung, bei der in der Regel zwei Techniken aus dem Erwartungshorizont geprüft werden (Tab. 1, linke Spalte), wurde für diese Studie eine alternative „Objective-Structured-Clinical-Exam-Prüfung“ (ToSkORL OSCE) mit einer Auswahl von neun Untersuchungstechniken inklusive der Endoskopie des Kehlkopfs und der Nasenhaupt- und -nebenhöhlen durchgeführt (Tab. 1, rechte Spalte). Die Studierenden erhielten schriftliche Anweisungen/Fragestellungen zur Durchführung der Untersuchungstechniken im Rahmen der strukturierten Prüfung. Drei Dozierende aus dem Studienteam bewerteten mit einem standardisierten Bogen anhand von fünfstufigen Likert-Skalen („trifft voll zu“ bis „trifft gar nicht zu“) den dargelegten theoretischen Hintergrund, die praktische Durchführung sowie eine Gesamtwertung für die jeweilige Untersuchungstechnik sowie das Ausmaß der notwendigen Hilfestellung zur korrekten Durchführung der jeweiligen Untersuchung. Die drei Dozierenden hatten Facharztniveau und mindestens 3 Jahre Erfahrung in der universitären Lehre. Bei den ersten drei ToSkORL-OSCE-Prüfungen wurde die Auswertemethodik gemeinsam durchgeführt, um nach einem gemeinsamen Standard zu prüfen und zu bewerten. Um eine Vergleichbarkeit mit den Selbsteinschätzungen der Studierenden zu ermöglichen, wurden die Untersuchungsblöcke ebenfalls anhand einer Likert-Skala bewertet.

Insgesamt 91 Studierende wurden in Gruppen aus zwei oder drei Studierenden gemeinsam geprüft. In einer Gruppe wurde jede der neun Untersuchungstechniken einmalig geprüft und hierbei durch eine(n) Studierende(n) als Untersucher(in) und an einem oder einer Studierenden als Patient(in) durchgeführt, mithin wurden alle neun Untersuchungstechniken je 42-mal durchgeführt. Die Tab. 2 zeigt Basisdaten für die Studierendenkohorte.

**Table 2:** 

Anzahl Studierende		*n* = 91
Alter		26 ± 4,1 Jahre
Geschlecht	Weiblich	46 (50,5 %)
	Männlich	44 (48,4 %)
	Keine Angabe	1 (1,1 %)
Fachsemester		8,9 ± 0,6
Famulaturen in HNO		4 (4,4 %)
Absolvierte HNO-Untersuchungskurse (max. 4)		3,4 ± 0,5

### Endpunkte

Für alle ausgewerteten Untersuchungstechniken ergaben sich auf diese Weise Selbst- und Fremdbewertungen. Die Fremdbewertung durch die Prüfenden erfolgte in Unkenntnis der Selbsteinschätzung der Studierenden sowie der weiteren erhobenen Daten. Eine vorhergehende Pseudonymisierung ermöglichte bei der späteren Auswertung die Zuordnung von Fremd- und Selbsteinschätzung. Neben der Auswertung der Selbst- und Fremdbewertungen wurde die jeweilige Differenz aus Fremd- und Selbstbewertung errechnet (Differenz Δ_Einschätzung_ = Wert_Fremd_ − Wert_Selbst_). Positive Werte ergeben daher eine Selbstüberschätzung der eigenen Fertigkeiten der Studierenden gegenüber der Fremdbewertung, negative Werte eine Selbstunterschätzung gegenüber der Fremdbewertung. Keine Differenz resultiert bei Übereinstimmung von Selbst- und Fremdeinschätzung.

Auf der Basis der Mittelwerte der Selbstbewertungen aller 91 Studierenden zu den neun Untersuchungstechniken erfolgte eine Zusammenfassung der Untersuchungstechniken in drei Gruppen mit unterschiedlichem wahrgenommenen Kompetenz‑/Anforderungsniveau.

### Statistische Analyse

Für die statistischen Analysen wurde SPSS in der Version 25.0.0.1 (IBM, Armonk/NY, USA) genutzt. Für Mittelwertvergleiche von mehr als zwei Gruppen erfolgten eine ANOVA und für Mittelwertvergleiche von zwei Gruppen t‑Tests. Zur systematischen Analyse von Einflussfaktoren auf die Differenzen von Selbst- und Fremdeinschätzung erfolgten multiple lineare Regressionsanalysen. Die Graphen wurden mit GraphPad Prism 8.4.3 (GraphPad, San Diego/CA, USA) erstellt.

### Interessenkonflikte und Ethikvotum

Die Autoren geben keine finanziellen oder persönlichen Verbindungen an, welche die Arbeit oder das Design dieses Projekts beeinflussen. Interessenkonflikte werden von allen AutorInnen verneint. Die Datenerhebung, -verarbeitung und -analyse erfolgte nach Einverständnis des lokalen Ethikkomitees (Ethikkommission der Medizinischen Fakultät der Ludwig-Maximilians-Universität, Votum 19-333) sowie in Einklang mit der Deklaration von Helsinki.

## Ergebnisse

### Die Einschätzung der eigenen Kompetenz von Studierenden in verschiedenen Untersuchungstechniken ist sehr variabel

**Figure 1:**
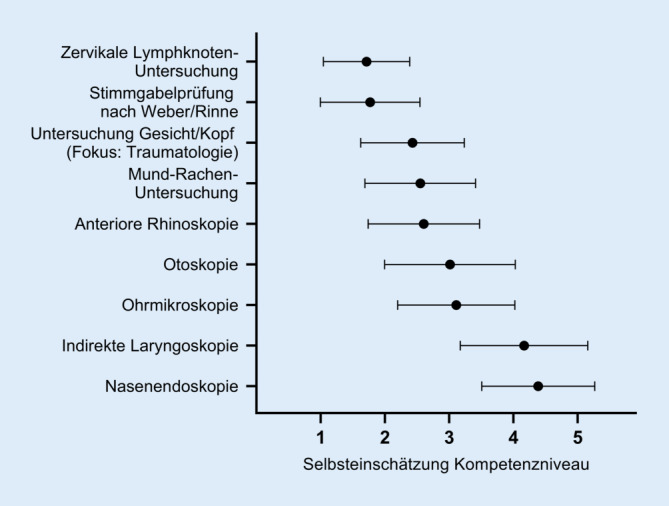

Sämtliche neun Einzel-Untersuchungseinheiten wurden von den Teilnehmern im Hinblick auf das subjektive Kompetenzniveau bewertet (Beherrschung der Untersuchung: „trifft voll zu“ = 1 bis „trifft nicht zu“ = 5). Die beste Selbsteinschätzung mit der niedrigsten Varianz zeigte sich bei der Untersuchung der Lymphknoten des Halses (Mittelwert: 1,71; σ^2^ = 0,45). Ein hohes Kompetenzniveau gaben die Teilnehmer mit einer geringen Varianz ebenfalls für die Untersuchung des Gesichts/Kopfs mit traumatologischem Fokus sowie für die Stimmgabelprüfung nach Weber und Rinne an. Ihre Kompetenz zur Durchführung der weiteren Untersuchungseinheiten schätzten die Teilnehmer bei höherer Varianz als geringer ein (Abb. 1). Abhängig von der Selbsteinschätzung zum Kompetenzniveau sowie der Schwierigkeit der Untersuchung erfolgte eine Gruppierung der Untersuchungseinheiten in „einfach“ (Untersuchung von zervikalen Lymphknoten, des Kopf/Gesichtsbereichs, Stimmgabelprüfung nach Weber/Rinne), „mittlere Schwierigkeit“ (Mund-Rachen-Inspektion, Otoskopie, anteriore Rhinoskopie) mit Einsatz einfacher Instrumente sowie „schwierig“ (Ohrmikroskopie, indirekte Laryngoskopie, Nasenendoskopie) mit Einsatz des speziell HNO-ärztlichen Instrumentariums.

### Studierende können ihre eigene Untersuchungskompetenz zumeist realistisch einschätzen

**Figure 2:**
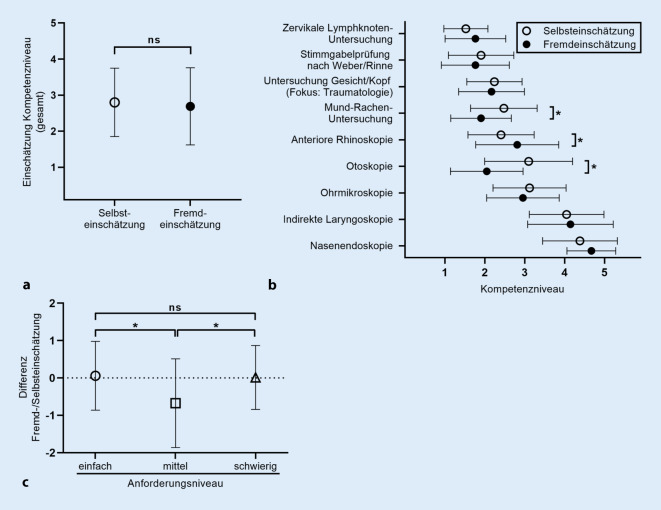

Die Durchführung der Untersuchungen wurde, wie beschrieben, von Dozierenden standardisiert bewertet, sodass ein direkter Vergleich dieser Bewertung mit den zuvor erhobenen Selbsteinschätzungen der Studierenden hinsichtlich ihres Kompetenzniveaus möglich war. Hierbei zeigte die Untersuchung mittels t‑Test (gepaarte Stichproben) in der Mehrzahl der Untersuchungen eine gute Übereinstimmung von Fremd- und Selbstbewertung. Der Gesamtmittelwert der Selbstbewertung lag bei 2,80 ± 0,95 (Standardabweichung), die Dozierendenbewertung bei 2,69 ± 1,07 (Standardabweichung). Eine signifikante Differenz zugunsten einer besseren Fremdbewertung war bei der Mund-Rachen-Inspektion sowie der Otoskopie erkennbar (Δ_Einschätzung_ = −0,57; *p* = 0,001 bzw. Δ_Einschätzung_ = −1,05; *p* < 0,001). Die Studierenden unterschätzten folglich ihr eigenes Kompetenzniveau in diesen Untersuchungen, wohingegen die Fremdeinschätzung bei der anterioren Rhinoskopie sowie der Nasenendoskopie signifikant beziehungsweise tendenziell schlechter ausfiel (Δ_Einschätzung_ = 0,40; *p* = 0,025 bzw. Δ_Einschätzung_ = 0,29; *p* = 0,050). Bei Gruppierung der Untersuchungseinheiten in die oben beschriebenen Gruppen „einfach“, „mittlere Schwierigkeit“ und „schwierig“ zeigte sich in der Varianzanalyse (ANOVA, Post-hoc-Test mittels Bonferroni-Korrektur), dass bei mittlerer Schwierigkeit die Differenz von Fremd- und Selbsteinschätzung signifikant höher war als bei einfachen sowie schwierigen Untersuchungen (*p* < 0,005). Die Ergebnisse sind in Abb. 2 zusammengefasst.

### Studierende mit hohem Interesse am Fachgebiet HNO-Heilkunde geben bessere Selbsteinschätzungen an, überschätzen sich jedoch eher

**Figure 3:**
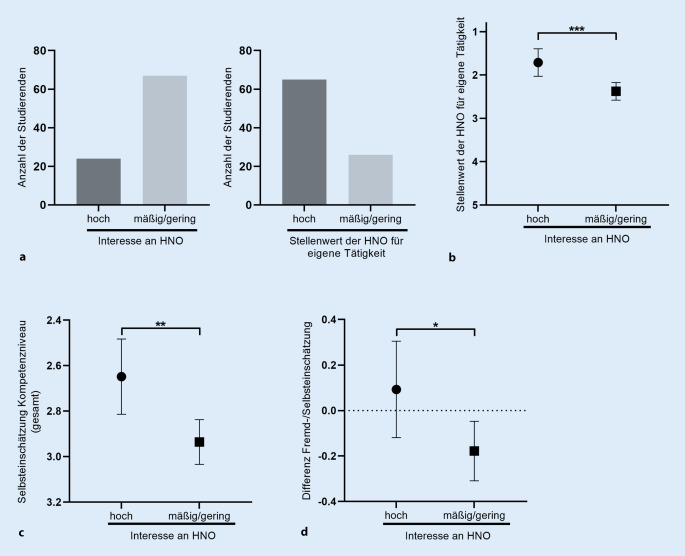

Im Folgenden wurde untersucht, ob sich die Ergebnisse von Studierenden mit hohem Interesse am Fachgebiet der Hals-Nasen-Ohren-Heilkunde (Summe der Likert-Skalenpunkte: 1–2) von denen der Studierenden mit mäßigem bis geringem Interesse (Summe der Likert-Skalenpunkte: 3–5) unterscheiden. 24 Studierende (26,4 %) gaben ein hohes Interesse am Fachgebiet an, wohingegen 67 Studierende (73,6 %) mäßiges oder geringes Interesse zeigten. Erstere hielten Kompetenzen in HNO-Heilkunde für signifikant wichtiger für ihre zukünftige berufliche Tätigkeit als die zweite Gruppe (1,71 vs. 2,37; *p* = 0,0008). Insgesamt hielten 65 (71,4 %) Studierende Kompetenzen in der HNO für ihre zukünftige ärztliche Tätigkeit für wichtig (Likert-Skala 1–2), wohingegen 26 (28,6 %) Studierende nur eine mäßige bis geringe Wichtigkeit hierin erkannten (Likert-Skala 3–4). Studierende mit hohem Interesse an der Hals-Nasen-Ohren-Heilkunde stuften ihre eigenen Untersuchungsfertigkeiten signifikant besser ein (2,65 vs. 2,94; *p* = 0,0035). Jedoch überschätzten sie ihre Kompetenzen auch eher als Studierende mit mäßigem bis geringem Interesse an der HNO-Heilkunde (Δ_Einschätzung_ 0,093 vs. −0,18; *p* = 0,033), wie in Abb. 3 gezeigt wird. Die Selbsteinschätzung der Kompetenzen in allen bewerteten Untersuchungseinheiten wurde hierfür miteinander verglichen.

### Die Selbsteinschätzungskompetenz der Studierenden ist weitgehend unabhängig von Alter und Geschlecht

Ergänzend untersuchten wir in einem multiplen linearen Regressionsmodell verschiedene unabhängige Variablen im Hinblick auf den Einfluss auf die Konkordanz von Selbst- und Fremdeinschätzung (Tab. 3). Es wurde das Alter der Studierenden, das Geschlecht und das Interesse an HNO-Heilkunde (hier in detaillierter intervallskalierter Abstufung 1–5: hoch bis niedrig) untersucht. Bei den als „schwer“ eingestuften Untersuchungstechniken (im Besonderen bei der Nasenendoskopie) war das Geschlecht ein Prädiktor für die Selbsteinschätzungsgüte, männliche Studierende neigten hier in absolut gesehen geringem Maß eher zu Selbstüberschätzung. Das Alter stellte sich als Prädiktor für eine hohe Konkordanz von Fremd- und Selbsteinschätzung bei der Nasenendoskopie dar, das Interesse an HNO-Heilkunde bei der Stimmgabeluntersuchung. In der überwiegenden Zahl der Untersuchungstechniken im Einzelnen als auch in der Zusammenfassung einzelner Untersuchungen nach Schwierigkeitsgrad oder Organgebiet war die Selbsteinschätzungsgüte unabhängig vom Alter oder dem Geschlecht der Studierenden (Tab. 3).

**Table 3:** 

		Unabhängige Variablen		
Untersuchungstechniken	*p* (Regressionsanalyse)	*p* (Alter)	*p* (Geschlecht)	*p* (Interesse an HNO)
Gesamt	0,161	0,516	0,066	0,379
Leicht	0,078	0,697	0,661	*0,012*
Mittelschwer	0,160	0,558	*0,026*	0,889
Schwer	**0,010**	**0,158**	***0,006***	**0,736**
Organgebiet äußerer Kopf-Hals-Bereich	0,119	0,176	0,488	0,068
Organgebiet Ohr	0,124	0,767	0,210	*0,046*
Organgebiet Mundhöhle/Rachen, Larynx	0,992	0,877	0,994	0,778
Organgebiet Nase und Nebenhöhlen	0,096	*0,038*	0,154	0,786
Lymphknoten	0,288	0,500	0,165	0,224
Stimmgabel	**0,016**	**0,060**	**0,321**	***0,004***
Äußerer Kopf	0,232	0,267	0,735	0,127
Mundhöhle, Rachen	0,273	0,328	0,169	0,162
Anteriore Rhinoskopie	0,452	0,170	0,592	0,363
Otoskopie	0,482	0,523	0,161	0,617
Ohrmikroskopie	0,139	0,051	0,097	0,841
Laryngoskopie	0,685	0,259	0,778	0,573
NNH-Endoskopie	**0,001**	***0,025***	***0,000***	**0,656**

Fett hervorgehoben in der Tabelle sind die Regressionsanalysen mit einem signifikanten Ergebnis in der statistischen Testung, jeweils dann noch kursiv hervorgehoben sind die bei diesen Regressionsanalysen signifikanten unabhängigen Variablen.

## Diskussion

Eine zunehmende Praxisorientierung des Medizinstudiums mit einer möglichst umfassenden und fundierten Ausbildung der Studierenden soll den langfristigen Erwerb von Kompetenzen in praktischen Fertigkeiten gewährleisten [11–14]. In der medizindidaktischen Forschung haben sich in diesem Zusammenhang insbesondere Studien bewährt, die anhand standardisierter Auswertemethoden die Selbst- und Fremdeinschätzung verglichen [5]. Diese umfassen in der Regel entweder Korrelationsanalysen oder Vergleichsanalysen verbundener oder unverbundener Datensätze aus Selbst- und Fremdeinschätzung, [5] In der Literatur finden sich verschiedene Studien, in welchen beispielsweise Abweichungen zwischen Selbst- und Fremdeinschätzung in der Einschätzung von Kompetenzen bei der Anamnese in der Zahnmedizin oder bei „objective structured clinical exams“ (OSCE) in der Pädiatrie ausgewertet wurden [15, 16]. Für die vorliegende Studie wurden neun Untersuchungskomplexe ausgewählt, die das Fachgebiet Hals-Nasen-Ohren-Heilkunde mit seinen Organgebieten breit und repräsentativ abbilden. Ähnliche Untersuchungskomplexe wurden bereits in deutschsprachigen Studien zur Lernkurve beim Erlernen der HNO-Spiegeluntersuchung beziehungsweise der Operationalisierung der HNO-Spiegeluntersuchung verwendet [9, 10]. In die Gesamtbewertung der Fremdeinschätzung flossen sowohl die Fähigkeiten der gebietsspezifischen Anamnese – somit theoretische Grundlagen – als auch praktische Fertigkeiten auf Basis der Durchführung der Untersuchung ein.

Neben einzelnen Fähigkeiten wurde auch der Einfluss von Geschlecht, Alter, Vorerfahrung und fachlichem Interesse auf eine bessere oder schlechtere Selbsteinschätzung erforscht. Aus einzelnen Studien dieser Art wird z. B. die Hypothese abgeleitet, dass weibliche Teilnehmer zu einer Unterschätzung ihrer Fähigkeiten neigen [5, 17]. In unserer Kohorte fanden sich keine Hinweise auf systematische Geschlechterunterschiede bei der Güte der Selbsteinschätzung. Auch das Studierendenalter spielte keine wesentliche Rolle, obwohl es innerhalb der Kohorte teils große Altersunterschiede gab, die sich gut durch die Zulassungskriterien zum Medizinstudium in Deutschland über „Hochschulstart“ (ehemals „ZVS“) erklären lassen. Insofern spiegelt das Studierendenalter auch nur bedingt den Faktor Erfahrung wider, der in Studien kontrovers als möglicher Einfluss auf die Güte der Selbsteinschätzung gilt, da sich alle 91 Studierenden unserer Kohorte mit maximal 2 Fachsemestern Unterschied am Ende des klinischen Studienabschnitts befanden [3, 5, 18, 19].

Ein besonders auffälliges Ergebnis dieser Studie war die Identifikation einzelner Untersuchungstechniken, die als besonders leicht beziehungsweise gut beherrscht empfunden und auch fremdbewertet wurden, und andere, die als schwieriger und deren Ausführung als schlechter beherrscht eingestuft wurden. Dies liegt insgesamt am wahrscheinlichsten an Spezifika des Curriculums im LMU-Humanmedizinstudiengang „MeCuM“ und dem vorhandenen Lehrkonzept im Rahmen der HNO-Bedside-Kurse: Grund für die besonders guten Einstufungen bei der zervikalen Lymphknoten-Untersuchung könnte die zu diesem Zeitpunkt bereits ein bis zwei Fachsemester zuvor abgelegte Prüfung zur Lymphknoten-Untersuchung am ganzen Körper sein. Die Stimmgabelprüfungen nach Weber und Rinne sind bereits Teil der vorklinischen Lehre im physiologischen Praktikum. Die traumatologische Untersuchung des Kopfs überschneidet sich ebenfalls mit Lehrinhalten der Unfallchirurgie. Otoskopie und Mund-Rachen-Inspektion könnten bereits im Rahmen der Famulatur oder Praktika in Allgemeinmedizin, Pädiatrie oder Neurologie geübt worden sein. Dies unterstreicht wiederum den Stellenwert der Erfahrung in praktischen Fertigkeiten und dem mehrfachen Befassen mit einer Untersuchungstechnik, welche entscheidend zu einer besseren Kompetenz und einer besseren Einschätzung der eigenen Kompetenz beitragen [5, 10, 11].

Im traditionellen Lehrkonzept der LMU München mit dem in Tab. 1 aufgeführten Erwartungshorizont für die praktische Mini-CEX-Prüfung bestand zudem keine Fokussierung auf die Endoskopie-Techniken, sodass diese im Rahmen unserer Kohorte als besonders schwer wahrgenommen und die Kompetenz vergleichsweise schlecht bewertet wurde. Andererseits korreliert die beobachtete Graduierung bei den verschiedenen Untersuchungskomplexen damit, in welchem Ausmaß spezielles Instrumentarium eingesetzt werden muss: Die Untersuchungen mit Hilfsinstrumenten wie dem Nasenspekulum oder Ohrtrichter wurden als schwieriger empfunden und Untersuchungen mit Winkeloptiken als besonders schwierig.

Bei den als mittelmäßig schwierig empfundenen Techniken zeigten sich die größten Abweichungen zwischen Selbst- und Fremdeinschätzung. Dieses Ergebnis weist auf eine trügerische Fehleinschätzung der Studierenden hin. Sowohl eine Über- als auch Unterschätzung der eigenen Fähigkeiten kann im ärztlichen Beruf leicht zu unzureichenden Untersuchungsergebnissen und letztlich zu Fehldiagnosen führen [20].

In unserer Kohorte stuften sich Studierende mit hohem Interesse am Fachgebiet Hals-Nasen-Ohren-Heilkunde besser ein und neigten zur Selbstüberschätzung, absolut betrachtet jedoch nur in einem geringen Ausmaß. Ein eher gegenteiliges Ergebnis, dass sich Studierende mit schlechteren Studiennoten überschätzten und sich solche mit besseren Noten unterschätzten, zeigte sich beispielsweise bei der Evaluation von Famulanten im Bereich der Gynäkologie [20]. Bei der Prüfung der Selbsteinschätzung verschiedener Skills in einem pädiatrischen OSCE fanden sich sowohl Skills, in denen die eigenen Fähigkeiten überschätzt, als auch Skills, in denen die eigenen Fähigkeiten unterschätzt wurden [16]. Eine generelle Selbstüberschätzung, die in anderen Kohorten für bestimmte Skills der Anamnese und Untersuchung gefunden wurde, konnte nicht festgestellt werden [15].

In der Literatur finden sich insgesamt starke Hinweise, dass trotz kleiner Abweichungen in Bezug auf einzelne Faktoren wie das Geschlecht oder das fachliche Interesse oder auf einzelne Skills die eigene Wahrnehmung oftmals gut mit der Einschätzung von außen korreliert [5]. Viele Studien geben Hinweise darauf, dass die Selbsteinschätzung mit wachsender (Berufs‑)Erfahrung zunehmend besser wird [4, 18, 21]. Trotzdem erscheint die Nutzung von Selbsteinschätzungen als Indikator für studentische Kompetenz beziehungsweise den Kompetenzerwerb weitgehend reliabel, insbesondere wenn man sich die eher geringe Tendenz zur Selbstüber- und -unterschätzung für einzelne Skills oder bestimmte Subkohorten bewusst macht [5, 12, 22, 23]. Für die chirurgische Weiterbildung in der Hals-Nasen-Ohren-Heilkunde konnte zudem gezeigt werden, dass die zeitnahe Erhebung der Daten nach der Phase des Kompetenzerwerbs wichtig ist [8].

Darüber hinaus lassen sich aus den Daten weitere Schlüsse folgern: die als mittelmäßig schwer empfundenen Untersuchungstechniken wie die Otoskopie, die anteriore Rhinoskopie und die Mundhöhlen-Rachen-Inspektion werden eher über- beziehungsweise unterschätzt, sodass in Zukunft der Fokus in der Lehre stärker auf diesen Untersuchungstechniken liegen sollte und zudem ergänzende Lehrangebote wie Refresher-Kurse zu einer nachhaltigen Verbesserung der praktischen Kompetenzen angeboten werden sollten. Dies konnte auch in der Arbeit von Polk et al. eindrücklich gezeigt werden, in dem mit der Durchführung eines stringenten repetitiven Lehrkonzepts während der Blockpraktikumswoche eine deutliche, signifikante Lernkurve beim Erlernen der HNO-Spiegeluntersuchung nachgewiesen werden konnte [10]. Ebenso gilt dies für die als schwierig empfundene Ohrmikroskopie und die Endoskopie-Techniken, für die eine hohe Unsicherheit bei den Studierenden besteht. Insgesamt weisen auch andere Studien auf Lücken beim Kompetenzerwerb während des Medizinstudiums hin [2, 24]. Strategien, die den Kompetenzerwerb unterstützen können, sind beispielsweise das regelmäßige Abfragen der selbst eingeschätzten Kompetenz in der Lernphase sowie das frühzeitige Einführen praktischer Lehrinhalte in das Medizinstudium [10, 11, 25]. Ebenso kann ein operationalisiertes Konzept, das die Vermittlung der HNO-Spiegeluntersuchung als Erlernen psychomotorischer Fähigkeiten standardisiert, helfen, das Niveau des Lernerfolgs der Studierenden und deren Akzeptanz des Lernkonzepts zu steigern [9].

Neuere Lernmethoden wie Blended Learning könnten ebenfalls hilfreich sein, den Kompetenzerwerb für Untersuchungstechniken des Kopf-Hals-Bereichs zu fördern. Eine Untersuchung aus dem Jahr 2017 ergab, dass solche Angebote, die Online-Inhalte mit Präsenzunterricht in einem abgestimmtem Lehrkonzept verbinden, im deutschsprachigen Raum relativ selten waren [26]. Die Evaluation eines solchen Konzepts wies vor allem auf die Bedeutung einer Aktivierung der Studierenden hin, um einen höheren Lernerfolg zu erreichen, selbst wenn solche Lernkonzepte trotz der Innovation nicht beliebter als klassische Lehrformen sein müssen [27, 28].

## Fazit für die Praxis

- Die Methodiken zur Messung von Kompetenz sind in der Regel sehr aufwendig, da hierfür für eine valide Auswertung meist sowohl Daten zur Selbst- als auch Fremdeinschätzung erhoben werden müssen.
- Jedoch erscheint die studentische Selbsteinschätzung zumindest in den meisten Fällen mit Fremdeinschätzungen durch Peers oder Lehrende stark zu korrelieren.
- Die vorliegende Studie unterstützt diese These, wobei eine Validierung mit einem Vergleich von Selbst- und Fremdeinschätzung weiterhin sinnvoll erscheint.
- Innerhalb einer Fakultät können zum Zweck der Erhebung eines Status quo oder zur Messung des Effekts einer neuen Lehrmethode zur Vermittlung praktischer Fähigkeiten durchaus auch die Erhebung der Selbsteinschätzung der Studierenden allein bereits sinnvoll und ausreichend sein.
- In der Zukunft muss in der universitären Hals-Nasen-Ohren-Heilkunde tendenziell mehr Wert auf die Vermittlung der speziellen Untersuchungstechniken des Kopf-Hals-Bereichs gelegt werden.
- Die Erhebung von Selbsteinschätzungen und die Korrelation mit Fremdeinschätzungen durch Lehrende können bei der Einführung neuer Lehrkonzepte ein wichtiges Werkzeug zur Auswertung sein.

## Supplementary Information





