# Supplementary material for: ToSkORL: self- and objective assessment of examination skills in the head and neck region
Source: HNO. 2021 Oct 7;70(4):295–303. [Article in German] doi: 10.1007/s00106-021-01097-y (PMC8964619; doi:10.1007/s00106-021-01097-y)
Supplement: Supplementary file 1 [file 106_2021_1097_MOESM1_ESM.pdf]

Mini-CEX Modul 4 (HNO)

1. **Allgemeines**

- Bitte geben Sie Ihr Alter in Jahren an: ☐☐
- Bitte geben Sie Ihr Geschlecht an: ☐ weiblich ☐ männlich ☐ divers ☐ k.A.
- Bitte wählen Sie aus der Liste Ihr aktuelles Fachsemester:
  ☐ 6 ☐ 7 ☐ 8 ☐ 9 ☐ 10 ☐ 11 ☐ 12 ☐ Ich weiß es nicht
- Haben Sie eine Famulatur/ein Praktikum in der Hals-Nasen-Ohrenheilkunde absolviert?
  ☐ ja ☐ nein
- Mein Interesse an der Veranstaltung und dem Fachgebiet Hals-Nasen-Ohrenheilkunde ist:
  sehr hoch ☐ ☐ ☐ ☐ ☐ sehr niedrig ☐ k.A.
- Wie viele Bedside-Kurse in der HNO haben Sie während des Blockpraktikums besucht?
  ☐ 1 ☐ 2 ☐ 3 ☐ 4

2. **Bitte geben Sie eine Einschätzung zum Grad Ihrer Kompetenzen und allgemeinen klinisch-praktischen Fähigkeiten zum jetzigen Stand Ihrer Ausbildung.**

Ich kann eine strukturierte allgemeine körperliche Untersuchung durchführen.	**Trifft voll zu**	☐☐☐☐☐	**Trifft gar nicht zu**	☐	**k.A.**
Ich halte Kompetenzen in der Diagnostik und Therapie von Erkrankungen des Fachgebiets der Hals-Nasen-Ohrenheilkunde als relevant für meine zukünftige ärztliche Tätigkeit.	**Trifft voll zu**	☐☐☐☐☐	**Trifft gar nicht zu**	☐	**k.A.**
Ich kann die Lymphknotenstationen des Halses strukturiert untersuchen (Inspektion, Palpation).	**Trifft voll zu**	☐☐☐☐☐	**Trifft gar nicht zu**	☐	**k.A.**
Ich kann den äußeren Kopf und das Gesicht untersuchen (Inspektion, Palpation, Perkussion).	**Trifft voll zu**	☐☐☐☐☐	**Trifft gar nicht zu**	☐	**k.A.**
Ich kann eine strukturierte klinische Untersuchung des Mund- und Rachen-Bereichs vornehmen	**Trifft voll zu**	☐☐☐☐☐	**Trifft gar nicht zu**	☐	**k.A.**
Ich kann eine indirekte Laryngoskopie mittels Endoskop durchführen.	**Trifft voll zu**	☐☐☐☐☐	**Trifft gar nicht zu**	☐	**k.A.**
Ich kann einen strukturierten Ohrbefund unter Zuhilfenahme eines Hand-Otoskops erheben.	**Trifft voll zu**	☐☐☐☐☐	**Trifft gar nicht zu**	☐	**k.A.**
Ich kann einen strukturierten Ohrbefund unter Zuhilfenahme eines Ohrmikroskops erheben.	**Trifft voll zu**	☐☐☐☐☐	**Trifft gar nicht zu**	☐	**k.A.**
Ich kann eine anteriore Rhinoskopie zur Untersuchung der Nasenhaupthöhle mittels Nasenspekulum durchführen.	**Trifft voll zu**	☐☐☐☐☐	**Trifft gar nicht zu**	☐	**k.A.**
Ich kann eine Endoskopie der Nasenhaupthöhle, des Nebenhöhlensystems und des Nasenrachens durchführen.	**Trifft voll zu**	☐☐☐☐☐	**Trifft gar nicht zu**	☐	**k.A.**
Ich kann eine Stimmgabelprüfung nach Weber und Rinne durchführen und interpretieren.	**Trifft voll zu**	☐☐☐☐☐	**Trifft gar nicht zu**	☐	**k.A.**
