# Supplementary material for: ToSkORL: self- and objective assessment of examination skills in the head and neck region
Source: HNO. 2021 Oct 7;70(4):295–303. [Article in German] doi: 10.1007/s00106-021-01097-y (PMC8964619; doi:10.1007/s00106-021-01097-y)
Supplement: Supplementary file 2 [file 106_2021_1097_MOESM2_ESM.pdf]

LMU

KLINIKUM
DER UNIVERSITÄT MÜNCHEN

CAMPUS GROSSHADERN
CAMPUS INNENSTADT

KLINIK UND POLIKLINIK FÜR HALS-NASEN-OHREN-HEILKUNDE

Mini-CEX Modul 4 (HNO)

1. **Allgemeines**

- Studierendenpärchen (Untersucher und Proband) für Mini-CEX bilden, keine weiteren Beobachter im Raum
- Aufteilen der Untersuchung (Gesamtuntersuchung durch 2 Studierende abgedeckt)
- Standardisierte Aufgaben vorlesen und Theorie/Durchführung bewerten
- Eingreifen/Hilfestellung, wenn Untersucher nicht in der Lage ist, die Untersuchung durchzuführen bzw. bevor er den Proband gefährdet

2. Evaluation klinisch-praktischer Fertigkeiten

Untersuchung des äußeren Halses (klinische Lymphknotenuntersuchung)

**Theorie:** Der Studierende kann die Halslevels (I-VI) und wichtige Kriterien zur Beschreibung von Raumforderungen benennen sowie das Vorgehen bei der Halsuntersuchung erläutern (strukturiertes Abtasten).	**Trifft voll zu**	☐☐☐☐☐	**Trifft gar nicht zu**
**Praxis**: Der Studierende kann die Untersuchung der Halslymphknoten strukturiert durchführen und ggf. getastete Lymphknoten präzise beschreiben.	**Trifft voll zu**	☐☐☐☐☐	**Trifft gar nicht zu**
War bei der Untersuchung **Hilfestellung** notwendig/musste **eingegriffen** werden?	☐ **Nein**	☐ **Ja, mündl. Hinweise**	☐**Ja, prakt. Hilfestellung oder Eingreifen**
**Gesamtbeurteilung**: Der Studierende kann die Untersuchung im Gesamten so durchführen, dass ein **valider Befund erhoben** werden kann.	**Trifft voll zu**	☐☐☐☐☐	**Trifft gar nicht zu**

Äußerer Kopf/Gesicht (Inspektion, Palpation, Perkussion)

**Theorie:** Der Studierende kann die Einzelschritte der Untersuchung des Gesichts-/Kopfbereichs erläutern (Inspektion, Palpation, Perkussion bei Trauma/V.a. Sinusitis).	**Trifft voll zu**	☐☐☐☐☐	**Trifft gar nicht zu**
**Praxis**: Der Studierende kann den äußeren Kopf-/Gesichtsbereich strukturiert untersuchen (Inspektion, Palpation (Nase, Orbitarahmen, Jochbogen, Maxilla, Zähne), Perkussion (NNH).	**Trifft voll zu**	☐☐☐☐☐	**Trifft gar nicht zu**
War bei der Untersuchung **Hilfestellung** notwendig/musste **eingegriffen** werden?	☐ **Nein**	☐ **Ja, mündl. Hinweise**	☐**Ja, prakt. Hilfestellung oder Eingreifen**
**Gesamtbeurteilung**: Der Studierende kann die Untersuchung im Gesamten so durchführen, dass ein **valider Befund erhoben** werden kann.	**Trifft voll zu**	☐☐☐☐☐	**Trifft gar nicht zu**

Mund-/Racheninspektion

**Theorie:** Der Studierende kann die Einzelschritte der Mund-/Racheninspektion erläutern (Stirnlicht, Mundspatel, Untersuchung Vestibulum oris, Zunge, Mundboden, Tonsillen, Speicheldrüsen).	**Trifft voll zu**	☐☐☐☐☐	**Trifft gar nicht zu**
**Praxis**: Der Studierende kann die Einzelschritte der die Einzelschritte der Mund-/Racheninspektion durchführen (korrekte Handhabung Stirnlicht/Mundspatel, Untersuchung Vestibulum oris, Zunge, Mundboden, Tonsillen, Speicheldrüsen).	**Trifft voll zu**	☐☐☐☐☐	**Trifft gar nicht zu**

War bei der Untersuchung **Hilfestellung** notwendig/musste **eingegriffen** werden? ☐ **Nein** ☐ **Ja, mündl. Hinweise** ☐ **Ja, prakt. Hilfestellung oder Eingreifen**

**Gesamtbeurteilung**: Der Studierende kann die Untersuchung im Gesamten so durchführen, dass ein **valider Befund erhoben** werden kann.	**Trifft voll zu**	☐ ☐ ☐ ☐ ☐	**Trifft gar nicht zu**

Indirekte Laryngoskopie

**Theorie:** Der Studierende kann die Einzelschritte der indirekten Laryngoskopie erläutern (insb. Positionieren des Patienten, Fassen der Zunge, Atmung durch den Mund) und die wichtigsten erkennbaren anatomischen Strukturen benennen.	**Trifft voll zu**	☐ ☐ ☐ ☐ ☐	**Trifft gar nicht zu**
**Praxis**: Der Studierende kann die Einzelschritte der indirekten Laryngoskopie durchführen (insb. Positionieren des Patienten, Fassen der Zunge, Atmung durch den Mund, vorsichtiges Einführen des Endoskops).	**Trifft voll zu**	☐ ☐ ☐ ☐ ☐	**Trifft gar nicht zu**

War bei der Untersuchung **Hilfestellung** notwendig/musste **eingegriffen** werden? ☐ **Nein** ☐ **Ja, mündl. Hinweise** ☐ **Ja, prakt. Hilfestellung oder Eingreifen**

**Gesamtbeurteilung**: Der Studierende kann die Untersuchung im Gesamten so durchführen, dass ein **valider Befund erhoben** werden kann.	**Trifft voll zu**	☐ ☐ ☐ ☐ ☐	**Trifft gar nicht zu**

Ohrmikroskopie/Otoskopie

**Theorie:** Der Studierende kann die Einzelschritte der Ohrmikroskopie erläutern (insb. Positionieren des Patienten, Zug an der Ohrmuschel, Auswahl/ Einführen des Ohrtrichters, Einstellen des Fokus).	**Trifft voll zu**	☐ ☐ ☐ ☐ ☐	**Trifft gar nicht zu**
**Praxis**: Der Studierende kann die Einzelschritte der Ohrmikroskopie durchführen (insb. Positionieren des Patienten, Zug an der Ohrmuschel, Auswahl/ Einführen des Ohrtrichters, Einstellen des Fokus).	**Trifft voll zu**	☐ ☐ ☐ ☐ ☐	**Trifft gar nicht zu**

War bei der Untersuchung **Hilfestellung** notwendig/musste **eingegriffen** werden? ☐ **Nein** ☐ **Ja, mündl. Hinweise** ☐ **Ja, prakt. Hilfestellung oder Eingreifen**

**Gesamtbeurteilung**: Der Studierende kann die Untersuchung im Gesamten so durchführen, dass ein **valider Befund erhoben** werden kann.	**Trifft voll zu**	☐ ☐ ☐ ☐ ☐	**Trifft gar nicht zu**
**Praxis (Otoskopie):** Der Studierende kann die Einzelschritte der Ohrmikroskopie durchführen (korrekte Kopfposition des Patienten, Zug an der Ohrmuschel, Einführen des Otoskops)	**Trifft voll zu**	☐ ☐ ☐ ☐ ☐	**Trifft gar nicht zu**
**Gesamtbeurteilung (Otoskopie):** Der Studierende kann die Untersuchung im Gesamten so durchführen, dass ein **valider Befund erhoben** werden kann.	**Trifft voll zu**	☐ ☐ ☐ ☐ ☐	**Trifft gar nicht zu**

Stimmgabelprüfung Weber/Rinne

**Theorie:** Der Studierende kann die Stimmgabelprüfung nach Weber und Rinne erläutern und eine einseitige Schallleitungs- und Schallempfindungsstörung differenzieren.	**Trifft voll zu**	☐ ☐ ☐ ☐ ☐	**Trifft gar nicht zu**
**Praxis**: Der Studierende kann die Stimmgabelprüfung nach Weber und Rinne korrekt durchführen (ausreichendes	**Trifft voll zu**	☐ ☐ ☐ ☐ ☐	**Trifft gar nicht zu**

Anschlagen der Stimmgabel, Positionieren der Stimmgabel, korrekte Patienteninstruktion).				
War bei der Untersuchung **Hilfestellung** notwendig/musste **eingegriffen** werden?	☐ **Nein**	☐ **Ja, mündl. Hinweise**	☐ **Ja, prakt. Hilfestellung oder Eingreifen**	
**Gesamtbeurteilung**: Der Studierende kann die Untersuchung im Gesamten so durchführen, dass ein **valider Befund erhoben** werden kann.	**Trifft voll zu**	☐☐☐☐☐	**Trifft gar nicht zu**	

Anteriore Rhinoskopie

**Theorie:** Der Studierende kann die Einzelschritte der anterioren Rhinoskopie erläutern (insb. Halten des Spekulums, Handhabung des Stirnlichts, korrektes Ein-/Ausführen des Spekulums) und erkennbare Strukturen benennen.	**Trifft voll zu**	☐☐☐☐☐	**Trifft gar nicht zu**	
**Praxis**: Der Studierende kann die Einzelschritte der anterioren Rhinoskopie durchführen (Stirnlicht einstellen, korrektes Halten des Spekulums, offenes Ein-/Ausführen, Vermeidung von Septumberührung).	**Trifft voll zu**	☐☐☐☐☐	**Trifft gar nicht zu**	
War bei der Untersuchung **Hilfestellung** notwendig/musste **eingegriffen** werden?	☐ **Nein**	☐ **Ja, mündl. Hinweise**	☐ **Ja, prakt. Hilfestellung oder Eingreifen**	
**Gesamtbeurteilung**: Der Studierende kann die Untersuchung im Gesamten so durchführen, dass ein **valider Befund erhoben** werden kann.	**Trifft voll zu**	☐☐☐☐☐	**Trifft gar nicht zu**	

Nasenendoskopie

**Theorie:** Der Studierende kann die Einzelschritte der Nasenendoskopie erläutern (insb. Vorbereitung mit Abschwellen/Betäuben, vorsichtiges Einführen) und erkennbare Strukturen benennen.	**Trifft voll zu**	☐☐☐☐☐	**Trifft gar nicht zu**	
**Praxis**: Der Studierende kann die Einzelschritte der Nasenendoskopie durchführen (vorsichtiges Vorschieben des Endoskops, Inspektion des mittleren Nasengangs und des Nasopharynx).	**Trifft voll zu**	☐☐☐☐☐	**Trifft gar nicht zu**	
War bei der Untersuchung **Hilfestellung** notwendig/musste **eingegriffen** werden?	☐ **Nein**	☐ **Ja, mündl. Hinweise**	☐ **Ja, prakt. Hilfestellung oder Eingreifen**	
**Gesamtbeurteilung**: Der Studierende kann die Untersuchung im Gesamten so durchführen, dass ein **valider Befund erhoben** werden kann.	**Trifft voll zu**	☐☐☐☐☐	**Trifft gar nicht zu**	
